# Medical Items

**Published:** 1894-10

**Authors:** 


### *MEDICAL ITEMS.*

THE twenty-first annual session of the Medical Society of Virginia will be held in Richmond, October 23d.

THE Mississippi Valley Medical Association will be held at Hot Springs, Ark., November 20th to 23d. The present indications are that this will be a large gathering.

BEGINNING with this issue our Department of the Eye, Ear, Nose, and Throat will be conducted by Dr. Dunbar Roy, Professor of Ophthalmology and Otology in the Southern Medical College.

DR. A. B. MILES, House Surgeon of the Charity Hospital, New Orleans, and Professor of Surgery in Tulane University (Medical Department), is dead. Dr. Miles was only forty-two years of age, but was a successful and prominent surgeon. Dr. Miles left $10,000 each to the Medical Department of Tulane University, the Charity Hospital, and the Hotel Dieu, all of New Orleans.

WANTED.—An association with a *thoroughly good* man, doing a large general and obstetrical practice. The advertiser has had an extensive *surgical and gynecological* experience in *two* of the largest and best hospitals in New York, and feels that such an association could be made *mutually advantageous.* Communications for this gentleman may be addressed to this JOURNAL

A WRITER in the *Texas Health Journal* says that Dr. J. C. LeHardy, of Savannah, has prepared several *monograms* relating to yellow fever. An article in a late Chicago *Medical Times* states that in cases of supposed pharyngitis sicca, it is important to make a careful examination of the *vulva* (uvula). In our own JOURNAL for last month the proof-reader made us say (p. 413) *vis medicatrix nostrum* (*naturæ*), which was an offence to *nature* quite unintentional. The Charlotte *Medical Journal* for last month speaks about the first *public* (pubic) symphyseotomy in America.

---

BY the indirect route of the *Progrès Medical* of Paris, we learn that a millionaire of Boston, Mr. Coates, who has arrived at the advanced age of eighty-three years without ever having taken medicine, has had the curious fancy of getting from druggists all the medicines which have been prescribed for him by doctors, carefully preserving the same. He had thus collected 1,900 bottles, 1,370 boxes of powders, and 870 boxes of pills. Our French contemporary might have added that Mr. Coates must have been a reader of Molière; When the great dramatist was once asked what he thought of his doctor, he replied : "Oh, when I am ill, I send for him. He comes ; we have a chat and enjoy ourselves. He prescribes ; I don't take his medicine, and am cured."

---

A LAY VIEW.—The following is from the *Philadelphia Record*: "Out of the forty-four States in the Union there are only sixteen in which a medical diploma of itself is no license to practice, and in which a State examination is required before legal permission to practice may be obtained. These sixteen States are Alabama, Arkansas, Florida, Maryland, Minnesota, Mississippi, New Jersey, New York, North Carolina, North Dakota, Pennsylvania, South Dakota, Texas, Utah, Virginia, and Washington. The ease with which bogus diplomas may be obtained in this and other countries, and the alarming prevalence and persistency of quackery, should awaken the legislatures of all the derelict commonwealths to the necessity for State supervision. Life ought not to be put in jeopardy through the ignorance of practitioners who are paid to cure, but who often blindly assist in swelling the death-rate.

